# Impact of a multidisciplinary clinical pathway on the management of spontaneous coronary artery dissection

**DOI:** 10.1007/s12471-026-02050-w

**Published:** 2026-05-18

**Authors:** Jin Al-Gully, Jessica P. Forouzanfar, Jose M. Montero-Cabezas, Jeroen Eikenboom, Paul L. den Exter, Sophie ter Haar, Bart Scheenstra, Melina C. den Haan, Frank van der Kley, J. Wouter Jukema, Brian O. Bingen, Ibtihal Al Amri

**Affiliations:** 1 Department of Cardiology, Leiden University Medical Center, Leiden, The Netherlands; 2 Women’s Heart Health Clinic, Leiden University Medical Center, Leiden, The Netherlands; 3 Department of Internal Medicine, Division of Thrombosis and Hemostasis, Leiden University Medical Center, Leiden, The Netherlands; 4 Basalt Rehabilitation, Leiden, The Netherlands; 5 Netherlands Heart Institute, Utrecht, The Netherlands

**Keywords:** Spontaneous Coronary Artery Dissection (SCAD), Acute Coronary Syndrome (ACS), Percutaneous Coronary Intervention (PCI), Tortuosity

## Abstract

**Aim:**

To evaluate the clinical impact of a dedicated multidisciplinary spontaneous coronary artery dissection (SCAD) care pathway compared with standard acute coronary syndrome management, focusing on safety, treatment patterns, clinical outcomes, and recurrence rates in patients with SCAD.

**Methods:**

In this retrospective observational cohort study, 117 SCAD patients were included: 63 managed within a SCAD-specific care pathway and 54 receiving standard care prior to or independent of its implementation. The SCAD pathway included protocolized angiographic diagnosis, conservative management when feasible, individualized medical therapy, screening for fibromuscular dysplasia (FMD) and systemic disorders, and SCAD-specific rehabilitation with structured follow-up. The primary endpoint was major adverse cardiovascular events (MACE) at 1‑year follow-up.

**Results:**

Patients in the SCAD pathway group were more often managed conservatively in the acute setting (76% vs. 24%, *p* < 0.001), had significantly lower rates of stent implantation (8% vs. 65%, *p* < 0.001), and were less frequently prescribed dual antiplatelet therapy (19% vs. 96%, *p* < 0.001). At 12-month follow-up, beta-blocker adherence was higher (76% vs. 41%, *p* < 0.001), aspirin use was lower (38% vs. 59%, *p* < 0.001), and recurrent SCAD occurred numerically less often but not statistically significantly (3% vs. 9%, *p* = 0.231). MACE rates were similar between groups, and no deaths occurred. FMD screening was more common in the pathway group (92% vs. 17%, *p* < 0.001), facilitating diagnosis and tailored long-term therapy.

**Conclusion:**

Implementation of a standardized SCAD care pathway was associated with a safe conservative approach, more targeted secondary prevention, improved beta-blocker adherence, and a trend toward fewer recurrent SCAD events. These findings support integration of SCAD-specific multidisciplinary care into routine clinical practice to improve diagnostic precision and long-term outcomes.

**Supplementary Information:**

The online version of this article (10.1007/s12471-026-02050-w) contains supplementary material, which is available to authorized users.

## Whats new?

- In the acute setting, a conservative approach was associated with comparable in-hospital safety outcomes and 1‑year MACE outcomes compared to standard ACS care
- Aspirin monotherapy appears safe in conservatively managed patients.
- A dedicated SCAD care pathway helps reduce overtreatment with DAPT, statins, and ACE inhibitors and improves beta-blocker adherence.
- Incorporating SCAD-specific multidisciplinary care into routine clinical practice may enhance diagnostic accuracy and long-term outcomes.

## Introduction

Growing awareness and research have improved recognition and management of spontaneous coronary artery dissection (SCAD). Previous studies support a conservative strategy in the acute setting, as most SCAD lesions heal spontaneously within weeks and percutaneous coronary intervention (PCI) carries a risk of iatrogenic dissection or hematoma propagation [1, 2]. While acute management strategies are more defined, optimal post-acute management and follow-up remain uncertain. Dual antiplatelet therapy (DAPT) may exacerbate intramural hematoma in SCAD, reducing intraluminal area, and emerging registry data suggest single antiplatelet therapy may reduce rates of major adverse cardiovascular events (MACE) [3]. In the absence of atherosclerosis or hyperlipidemia, statins are unlikely beneficial, as they do not target the underlying non-atherosclerotic pathophysiology and lack evidence of benefit in reducing recurrence. Identification and management of precipitating and predisposing conditions are critical for individualized management strategies. Retrospective data further suggest a potential protective role of beta-blockers in preventing SCAD recurrence, although randomized evidence is lacking [3].

To address heterogeneity of SCAD management, a standardized SCAD-specific clinical care pathway was implemented at our tertiary center. This initiative aligns with emerging evidence that multidisciplinary care models may enhance diagnostic accuracy and consistency in management across the SCAD care continuum [4]. While disease-specific clinical pathways have improved outcomes in other cardiovascular conditions, their impact on clinical outcomes in SCAD has not yet been systematically evaluated. This study evaluates whether implementation of a structured SCAD care pathway improves in-hospital and long-term clinical outcomes compared to standard care.

## Methods

### Study population

This retrospective observational cohort study included two groups of female SCAD patients. The first group comprised consecutive patients enrolled in an ongoing SCAD registry at our tertiary care center, managed according to a standardized SCAD care pathway implemented since July 2020. The second group included SCAD patients identified retrospectively from a historical cohort previously described, involving consecutive female patients aged ≤ 55 years who presented with acute coronary syndrome (ACS) [2]. Two cohorts were defined: 1) the SCAD pathway group and 2) the standard care group, consisting of patients treated prior to pathway implementation or in whom SCAD was not initially recognized at coronary angiography and were therefore treated according to contemporary ACS protocols. Patients with fatal cardiogenic shock at presentation were excluded. Patients in the standard care group were included between January 2012 and June 2020, whereas patients treated within the SCAD care pathway were included from July 2020 until December 2023. Details of angiographic diagnosis and inclusion criteria have been previously reported [2]. Data collection followed institutional ethics approval.

### Specialized SCAD clinical care pathway

All patients presented with ACS and were treated according to contemporary ACS guidelines [5].

**Fig. 1:**
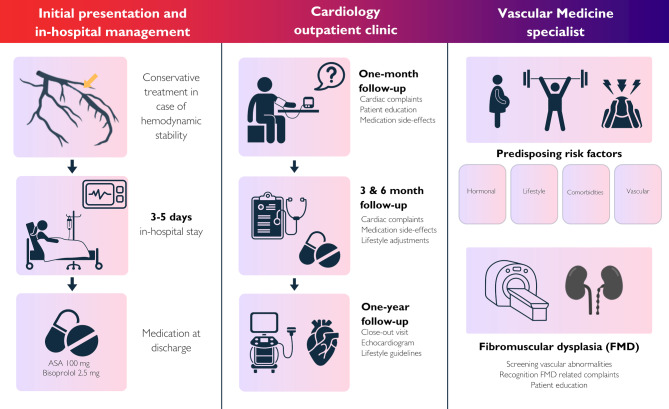
*SCAD care pathway. *Overview of the clinical pathway for SCAD patients, including prolonged in-hospital stay, scheduled cardiology follow-up and parallel referral to the vascular medicine specialist for screening of predisposing factors such as fibromuscular dysplasia (FMD)

SCAD was diagnosed during coronary angiography and classified according to the Yip-Saw criteria [6, 7]. In case of diagnostic uncertainty, intracoronary imaging was performed [7]. Standard care patients were managed according to the general ACS protocols in place at the time [8]. Consistently, these patients typically received DAPT for up to one year, lipid-lowering therapy, beta-blockers, and angiotensin-converting enzyme (ACE) inhibitors. In contrast, patients in the SCAD pathway group received standardized, protocol-based care initiated at the time of SCAD diagnosis during initial coronary angiography (Fig. 1). Angiography was modified to minimize contrast use and catheter manipulation, and procedures were terminated early after diagnosis [9]. Conservative management was applied in TIMI 2–3 flow without ischemia, while PCI was reserved for TIMI < 1, ongoing ischemia, high-risk anatomy, or hemodynamic instability [9]. Intracoronary imaging of non-culprit vessels assessed coexisting atherosclerosis, and vascular screening included carotid, renal, and iliofemoral arteries to detect FMD [10]. SCAD pathway patients were monitored in-hospital for 72–120 h with continuous telemetry (vs. 24–48 h standard care group). This extended observation period was implemented to support a non-interventional coronary management strategy, allowing for early detection of arrhythmias, dynamic ST-segment changes, and potential progression of the hematoma. When no percutaneous intervention was performed, patients in the SCAD pathway group were prescribed acetylsalicylic acid (ASA) monotherapy for up to one year. In cases of FMD or coexisting coronary atherosclerosis, indefinite ASA therapy was recommended [3, 11]. Statins were only initiated in patients with documented atherosclerosis or hypercholesterolemia. Beta-blockers were initiated and continued long-term in most patients unless contraindicated. This strategy was based on observational data suggesting a possible association with lower recurrence rates, although definitive evidence supporting routine lifelong therapy is lacking. [12].

**Fig. 2:**
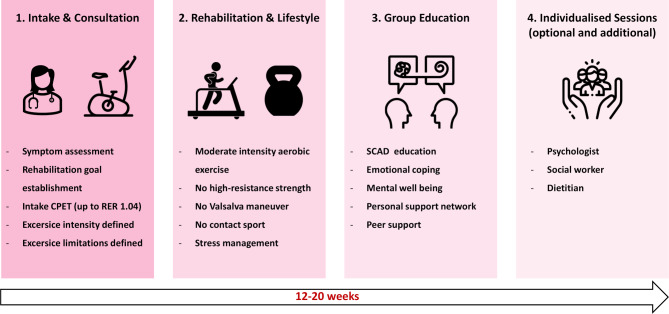
*SCAD-specific rehabilitation. *Overview of a tailored rehabilitation program for patients with SCAD, including an initial consultation with the rehabilitation cardiologist, moderate-intensity exercise sessions, lifestyle and stress management guidance and multidisciplinary group education. Optional individual support is provided by the psychologist, social workers, and dieticians. Duration: 12–20 weeks

SCAD patients managed within the specialized pathway were followed for at least one year in a dedicated SCAD outpatient clinic by both an interventional cardiologist and a vascular internist specialized in SCAD care. Follow-up included in-person visits at 1 and 12 months, and interim teleconsultations at 3 and 6 months. These consultations focused on education, investigation of potential precipitating or coexisting conditions, and individualized adjustment of medical therapy and symptom management. Risk profiling included assessment of traditional cardiovascular risk factors as well as precipitating factors associated with SCAD, being intense Valsalva maneuver, severe emotional stress, recreational drug use, and/or extreme physical exertion prior to the ACS event [13]. Emotional stress was defined as an acute psychological or affective trigger. Further investigation of underlying factors included recent reproductive hormonal changes (e.g., menopause, pregnancy, or hormone therapy). Investigation of autoimmune and connective tissue disorders was performed when clinically suspected. In patients who did not undergo angiographic screening for FMD at the time of index angiography, total-body computed tomography was performed. Genetic testing was considered in patients with recurrent or familial SCAD, vascular abnormalities or clinical features suggestive of inherited syndromes amenable to established genetic panels. Furthermore, SCAD patients were referred to a personalized cardiac rehabilitation program including an intensity-restricted cardiopulmonary exercise test (CPET) and follow-up with a physiotherapist. Exercise training was tailored individually based on baseline CPET results. Patients were encouraged to engage in regular physical activity while avoiding Valsalva maneuvers, high-intensity aerobic or resistance training, and extreme head and neck positions (Fig. 2). Multidisciplinary group sessions focused on education, peer support, and emotional processing, with special attention to the psychosocial consequences of the disease. Upon indication, individual consultations with other rehabilitation team members—for example, a psychologist or dietitian were offered [14–16].

**Fig. 3:**
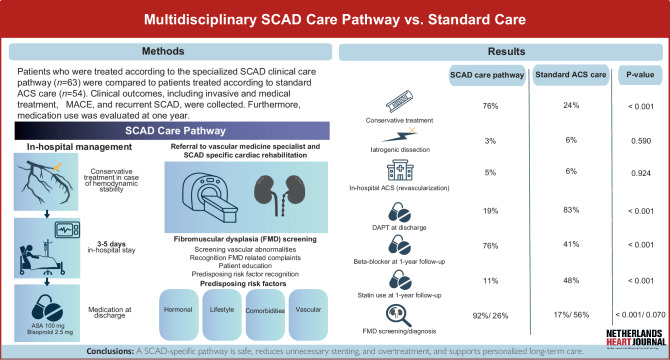
*Graphical abstract. ACS* Acute Coronary Syndrome, *ASA* Acetylsalicylic Acid, *SCAD* Spontaneous Coronary Artery Disease, *FMD* Fibromuscular Dysplasia, *DAPT* Dual Antiplatelet Therapy

All SCAD pathway patients were discussed in a multidisciplinary team involving the aforementioned specialists with input from other specialties (e.g., neurology, gynecology) when indicated for a patient-tailored care plan.

In both groups, patients were referred back to their general practitioner for continued cardiovascular risk management after one year if they were free of angina equivalents.

### Clinical data collection

Clinical, demographic, laboratory, angiographic, and electrocardiographic data were extracted from the Leiden University Medical Center (LUMC) electronic patient file. Chest pain characteristics were categorized as pressing, sharp, burning, and/or cramping [5]. ACS was defined according to the definitions provided by the European Society of Cardiology guidelines [5]. Stroke was defined as an episode of acute neurological dysfunction caused by ischemia or hemorrhage, persisting ≥ 24 h or until death [17, 18]. Cardiogenic shock was classified according to the classification of the Society for Coronary Angiography and Interventions [19].

### Follow-up and outcome measures

MACE was defined as a composite of all-cause mortality, ACS with or without required revascularization, stroke, bleeding requiring intervention, heart failure requiring hospitalization, and cardiogenic shock [20]. SCAD extension was also recorded and defined as the angiographic extension of a previously dissected coronary segment [9]. Both scheduled and unscheduled events were systematically recorded, including emergency department visits for chest pain or angina equivalents, medication-related adverse effects leading to dose adjustments, changes in medication class, or discontinuation, as well as repeat diagnostic coronary angiography. Post-index event comorbidities potentially linked to SCAD, including connective tissue and inflammatory disorders, FMD, and hypothyroidism, were also recorded. Recurrent SCAD was recorded and confirmed through clinical angiographic review.

### Study endpoints

This study aimed to evaluate the association between the implementation of a standardized SCAD-specific clinical care pathway and both in-hospital and long-term clinical outcomes, compared to patients managed prior to its adoption or independently of the pathway due to diagnostic uncertainty. The primary endpoint was the occurrence of MACE at 1‑year follow-up. Secondary endpoints include procedural treatment and complications, in-hospital MACE, medication at discharge, medication use at 1‑year follow-up, and occurrence of all-cause death and recurrent SCAD.

### Statistical methods

Data are presented as mean ± SD or median (IQR) for continuous variables and as counts and proportions for categorical variables. Normally distributed continuous variables were compared using *t*-test, while non-normally distributed variables were compared using the Mann-Whitney U test. Categorical variables were compared with the Chi-squared test or Fisher’s exact test. Independent predictors of SCAD were analyzed with multivariable models using a backward stepwise selection. Variables with a *p*-value were initially included. *P*-values < 0.05 were considered to indicate statistical significance. Statistical analysis was performed with the SPSS software (IBM SPSS version 25).

## Results

During a median follow-up of 12.1 months (IQR 11.7–22.9), 117 patients (mean age 50.6 ± 9.2 years) were included (54% SCAD pathway, 46% standard care). Baseline characteristics are detailed in Tab. 1.

**Table 1:** Baseline characteristics

	Total (*n* = 117)	SCAD pathway (*n* = 63)	Standard care (*n* = 54)	*P*-value
Age, y, mean ± SD	50.6 ± 9.2	53.0 ± 10.4	47.9 ± 6.6	0.002
Body mass index, kg/m^2^, mean ± SD	26.7 ± 5.1	27.5 ± 5.7	25.7 ± 4.2	0.061
Current smoker (%)	25 (21.4)	9 (14.3)	16 (29.6)	0.048
Previous smoker (%)	28 (23.9)	17 (27.0)	11 (20.4)	0.376
Hypertension (%)	40 (34.2)	20 (31.7)	20 (37.0)	0.589
Diabetes Mellitus type 2 (%)	2 (1.7)	1 (1.6)	1 (1.8)	0.931
Dyslipidemia (%)	23 (19.7)	18 (28.6)	5 (9.3)	0.007
History of CVA (%)	2 (1.7)	1 (1.6)	1 (1.8)	0.912
History of TIA (%)	3 (2.6)	1 (1.6)	2 (3.7)	0.652
Anxiety (%)	6 (5.1)	4 (6.3)	2 (3.7)	0.518
Depression (%)	12 (10.3)	9 (14.3)	3 (5.6)	0.121
Hypothyroidism (%)	6 (5.1)	4 (6.3)	2 (3.7)	0.102
Migraine (%)	22 (18.8)	17 (27.0)	5 (9.3)	0.833
History of chest pain (%)	29 (24.8)	15 (23.8)	14 (25.9)	0.777
Previous ACS (%)	8 (6.8)	4 (6.3)	4 (7.4)	0.839
Previous SCAD (%)	5 (4.3)	2 (3.2)	3 (5.6)	0.538
Previous PCI (%)	3 (2.6)	0	3 (5.6)	0.060

*CVA* Cerebrovascular Accident, *TIA* Transient Ischemic Attack, *ACS* Acute Coronary Syndrome, *SCAD* Spontaneous Coronary Artery Disease, *PCI* Percutaneous Coronary Intervention

Variables are either presented as mean ± SD or as *n* (%)

Within the standard care group, only 29.6% of patients were initially diagnosed as SCAD. The majority were misclassified as coronary thrombosis or atherosclerotic plaque (55.6%), coronary spasm (9.3%), or as having no angiographic abnormalities (5.6%) at the time of coronary angiography. All patients treated in the SCAD pathway group had a correctly diagnosed SCAD. Management differed substantially, with more frequent conservative treatment and less stent implantation in the SCAD pathway group (Tab. 2), while angiographic complications were similar.

**Table 2:** Angiographic and procedural features

	Total (*n* = 117)	Treated in SCAD care pathway (*n* = 63)	Not treated in SCAD care pathway (*n* = 54)	*P*-value
Iatrogenic dissection (%)	5 (4.3)	2 (3.2)	3 (5.6)	0.590
Invasive treatment (%)	56 (47.9)	15 (23.8)	41 (75.9)	**<** 0.001
– Intramural hematoma expansion (%)	19 (33.9)	4 (26.7)	15 (36.6)	0.488
– Drug-eluting stent (%)	40 (71.4)	5 (33.3)	35 (64.8)	**<** 0.001
– Guidewire only PCI (%)	7 (12.5)	5 (33.3)	2 (4.9)	0.336
– ≥ 3 stents in culprit (%)	15 (26.8)	2 (13.3)	13 (31.7)	0.858
– ≥ 2 stents in culprit (%)	29 (51.8)	4 (26.7)	25 (61)	0.813
*Culprit SCAD (%)*				
– RCA	20 (17.1)	13 (20.6)	7 (13.0)	0.272
– LAD	60 (51.3)	28 (44.4)	32 (59.3)	0.110
– LCx	35 (29.9)	21 (33.3)	14 (25.9)	0.383
– LM	2 (1.7)	1 (1.6)	1 (18.5)	0.912
Multivessel SCAD (%)	5 (4.3)	3 (4.8)	2 (37.0)	0.778
*Type of SCAD (%)*				
– Type 1	9 (7.7)	3 (4.8)	6 (11.1)	0.199
– Type 2a	37 (31.6)	16 (25.4)	21 (38.9)	0.118
– Type 2b	36 (30.8)	28 (44.4)	8 (14.8)	< 0.001
– Type 3	11 (9.4)	4 (6.3)	7 (13.0)	0.222
– Type 4	24 (20.5)	12 (19.0)	12 (22.2)	0.672
Intracoronary imaging culprit vessel (%)	21 (17.9)	11 (17.5)	10 (18.5)	0.882
– IVUS	19 (16.2)	9 (14.3)	10 (18.5)	0.156
– OCT	2 (1.7)	2 (3.2)	0	0.156

Variables are presented as *n* (%)

*DES* drug-eluting stent, *IVUS* intravascular ultrasound, *LAD* left anterior descending coronary artery, *LCx* left circumflex coronary artery, *LM* left main coronary artery, *OCT* optical coherence tomography, *PCI* percutaneous coronary intervention, *RCA* right coronary artery, *SCAD* spontaneous coronary artery dissection

In-hospital outcomes were comparable between groups (Tab. 3), although recurrent ACS without revascularization occurred more frequently in the SCAD pathway group. At discharge, patients in the pathway group received less DAPT, statins, and ACE inhibitors, with similar beta-blocker use. At 1 year, beta-blocker use was higher and acetylsalicylic acid use lower in the pathway group, without differences in left ventricular function, ACS recurrence, or angina-related emergency visits.

**Table 3:** In-hospital and 1‑year clinical outcomes of the SCAD clinical pathway

Follow-up	Total (*n* = 117)	SCAD pathway (*n* = 63)	Standard care (*n* = 54)	*P*-value
Admission length (days, IQR)	3 (2–5)	4 (3–5)	2 (2–4)	0.005
*Medical treatment at discharge*				
ASA (%)	112 (95.7)	59 (93.7)	53 (98.1)	0.231
P2Y12 inhibitor (%)	67 (57.3)	14 (22.2)	53 (98.1)	**<** 0.001
DAPT (%)	64 (54.7)	12 (19.0)	52 (96.3)	**<** 0.001
Beta blocker (%)	103 (88.0)	58 (92.1)	45 (83.3)	0.147
Statin (%)	81 (69.2)	30 (47.6)	51 (94.4)	**<** 0.001
Other cholesterol-lowering medication (%)	3 (2.6)	3 (4.8)	0	0.107
ACE-inhibitor (%)	81 (69.2)	35 (55.6)	46 (85.2)	**<** 0.001
*In-hospital follow-up*				
≥ 1 In-hospital re-CAG (%)	7 (6.0)	4 (6.3)	3 (5.6)	0.590
≥ 1 In-hospital ACS (no revascularization) (%)	4 (3.4)	4 (6.3)	0	0.026
≥ 1 In-hospital ACS requiring revascularization (%)	6 (5.1)	3 (4.8)	3 (5.6)	0.924
≥ 1 SCAD extension	6 (5.1)	3 (4.8)	3 (5.6)	0.843
Cardiogenic shock requiring mechanical support (%)	2 (1.7)	1 (1.6)	1 (1.8)	0.960
– 1 Bleeding event requiring intervention (%)	3 (2.6)	2 (3.2)	1 (1.8)	0.652
*1‑year follow-up*				
LVEF good > 50% (%)	52 (44.4)	26 (41.3)	26 (48.1)	0.603
LVEF mildly reduced > 30% (%)	11	4	7	0.379
LVEF moderately reduced ≤ 3 0% (%)	0	1	0	0.298
≥ 1 Hospital/ER visits chest pain, no indication of ACS (%)	17 (14.5)	7 (11.1)	10 (18.5)	0.329
≥ 1 ACS event, all (%)	7 (6.0)	2 (3.2)	5 (9.3)	0.274
≥ 1 ACS events (hospitalization, no revascularization) (%)	5 (4.3)	2 (3.2)	3 (5.6)	0.750
≥ 1 ACS requiring revascularization (%)	2 (1.7)	0	2 (3.7)	0.172
Experienced Beta-blocker-related side-effects (%)	27 (23.1)	10 (15.9)	17 (31.5)	0.046
Experienced ASA-related side-effects (%)	3 (2.6)	2 (3.2)	1 (1.8)	0.652
Experienced P2Y12 inhibitor-related side-effects (%)	5 (4.3)	2 (3.2)	3 (5.6)	0.526
FMD screening (%)	67 (57.3)	58 (92.1)	9 (16.7)	**<** 0.001
– FMD diagnosis (%)	20 (29.8)	15 (25.9)	5 (55.6)	0.070
*Medication at 1‑year FU*				
ASA (%)	56 (47.9)	24 (38.1)	32 (59.3)	**<** 0.001
P2Y12 inhibitor (%)	9 (7.7)	4 (6.3)	5 (9.3)	0.419
Beta blocker (%)	70 (60.0)	48 (76.2)	22 (40.7)	**<** 0.001
Statins (%)	33 (28.2)	7 (11.1)	26 (48.1)	**<** 0.001
DAPT (%)	3 (2.6)	0	3 (5.6)	0.058
*Median follow-up of 12.1 months (11.7–22.9)*				
Death (%)	0	0	0	n/a
≥ 1 recurrent SCAD (%)	7 (6.0)	2 (3.2)	5 (9.3)	0.231

*ASA* Acetylsalicylic acid, *P2Y12 inhibitor* P2Y purinoceptor 12 inhibitor, *DAPT* Dual Antiplatelet Therapy, *LMWH* Low Molecular Weight Heparin, *ACE-inhibitor* Angiotensin Converting Enzyme inhibitor, *ACS* Acute Coronary Syndrome, *SCAD* Spontaneous Coronary Artery Disease, *CAG* Coronary Angiography, *ER* Emergency Room, *FMD* Fibromuscular Dysplasia

Variables are either presented as median (Q1–Q3) or as *n* (%)

Screening for fibromuscular dysplasia was performed more frequently in the pathway group (Tab. 3; Electronic Supplementary Material Table S1). Additional diagnoses included Ehlers-Danlos syndrome (2%), hypothyroidism (6%), and a variant of uncertain significance in *FBN1* in one patient. A potential SCAD trigger was identified in 59% of patients.

No deaths occurred during follow-up. Recurrent SCAD was infrequent and did not differ significantly between groups.

## Discussion

Implementation of a standardized, multidisciplinary clinical care pathway for patients with SCAD altered acute management, diagnostic evaluation, and outpatient care. The principal findings can be summarized as follows (Fig. 3): 1) Patients in the SCAD pathway group were more often treated conservatively during emergent catheterization, which was not associated with a higher rate of in-hospital complications, nor diminished LV function at 1‑year follow up; 2) Patients in the SCAD pathway group were less often treated with medications of limited benefit in the absence of atherosclerotic ischemic heart disease; 3) Adherence to beta-blocker therapy was significantly higher at 1‑year in the SCAD pathway; 4) Systematic screening yielded a potential predisposing condition or trigger in the vast majority of patients within the SCAD pathway; 5) The incidence of recurrent SCAD after 1‑year was low and did not significantly differ between groups.

A growing body of evidence supports conservative management in hemodynamically stable SCAD patients, particularly in the absence of ongoing ischemia or high-risk anatomy [2, 21]. In our SCAD pathway cohort, conservative management was associated with a lower rate of intramural hematoma expansion and stent implantation compared to patients outside the pathway. The fact that neither the rate of in-hospital ACS requiring revascularization, nor the rate of in-hospital MACE, nor the LV function at 1‑year was altered by conservative management, underscores the concept that myocardial recovery is not compromised by avoiding potentially harmful PCI in stable SCAD presentations. The incidence of iatrogenic catheter-induced dissection did not differ significantly between groups, suggesting that coronary angiography can be performed safely in SCAD.

Among non-invasively managed patients, episodes of recurrent angina and transient ST-segment changes during hospitalization, likely reflecting residual coronary stenosis, were observed more frequently in the SCAD pathway group. All of these events resolved with medical therapy alone, and none required revascularization. This higher detection rate likely reflects prolonged in-hospital telemetry monitoring (72–120 h), which increases sensitivity for transient ischemic changes, as well as the intentional adoption of a conservative management strategy, in which dynamic residual coronary obstruction is expected to resolve without repeat intervention. Importantly, this observation should therefore not be interpreted as a signal of adverse early instability, but rather as supportive of the safety of conservative management in appropriately selected SCAD patients. The notion that ST changes are associated with a 2-fold increase in mortality risk and angina with ST changes was observed in 6% of the patients in the SCAD pathway group(while these events may have been missed in the standard care pathway group), which supports protocolized prolonged in-hospital stay in SCAD-related ACS [22]. The high proportion of initially unrecognized or misclassified SCAD in the standard care group likely had a major impact on acute revascularization decisions, underscoring the importance of early diagnostic accuracy.

The SCAD pathway enabled tailored therapy, reducing unnecessary DAPT and statin use while increasing beta-blocker adherence [7, 23]. The proportion of patients discharged on DAPT exceeded the proportion of patients receiving a coronary stent, reflecting the fact that DAPT was routinely prescribed after any coronary intervention, including balloon angioplasty, even when no stent was implanted. Higher reported side effects of beta-blockers in standard care likely reflect dosing and lack of titration rather than true intolerance, as pathway-based therapy was initiated at lower doses and adjusted during follow-up.

Evidence regarding the role of beta-blockers in preventing SCAD recurrence is limited to observational studies with inconsistent findings. Current consensus statements do not support routine lifelong therapy for recurrence prevention, and management should be individualized pending prospective data.

A key strength of the SCAD pathway is the integration of psychosocial support, tailored cardiac rehabilitation, and longitudinal follow-up within a dedicated outpatient clinic. Patients underwent systematic assessment for potential triggers and predisposing conditions adjusting the risk of SCAD recurrence (depending on treatment) or bearing a substantial risk of SCAD-unrelated morbidity, and as such, convey important consequences for clinical treatment. Within this latter context, the prevalence of FMD among SCAD patients is of particular interest. The observed prevalence of FMD in our cohort (25.9%) was lower than that reported in several prior SCAD registries (37–72%) [12, 24, 25]. This discrepancy is most plausibly attributable to differences in screening strategies, as we applied a systematic, non-targeted vascular imaging approach to all patients, irrespective of clinical pre-test probability. In contrast, studies reporting higher FMD prevalences often relied on selective imaging triggered by phenotypic features, which may introduce referral bias. Heterogeneity in imaging protocols, vascular bed coverage, and diagnostic criteria across studies further complicates direct comparison of reported prevalence rates. If FMD was confirmed, long-term aspirin was prescribed to mitigate thrombotic risk [11]. The presence of screened patients in the standard care group can largely be explained by the transitional period during pathway implementation. Importantly, aspirin was more often discontinued in the SCAD pathway group, reflecting more precise diagnostic classification and reduced exposure to unnecessary antiplatelet therapy. These findings underscore the clinical value of the screening for predisposing arteriopathies to guide long-term management and reduce bleeding risk.

Recurrent SCAD occurred in a small number of patients (*n* = 7), predominantly in the standard care group. While not statistically significant, this numerical trend may indicate a protective effect of the SCAD pathway, potentially mediated by improved beta-blocker adherence and structured lifestyle and rehabilitation strategies. This observation remains exploratory and warrants confirmation in prospective studies.

The observed clinical impact of the SCAD pathway is unlikely to be attributable to a single intervention in isolation. Rather, benefit appears to arise from the integrated combination of conservative management, tailored pharmacotherapy, structured follow-up, and rehabilitation.

Multidisciplinary care models have been widely adopted in heart failure, oncology, and complex surgical care. Our results suggest the potential value of a multidisciplinary SCAD program, addressing critical gaps in traditional ACS management by ensuring that SCAD-specific clinical and psychosocial needs are systematically addressed through the process.

## Limitations

Limitations include the retrospective single-center design, small sample size, potential information bias, and limited generalizability due to an exclusively female cohort.

## Conclusions

Implementation of a dedicated SCAD care pathway was associated with comparable in-hospital safety and 1‑year MACE, reduced overtreatment, improved beta-blocker adherence, and fewer hospital visits for angina without ACS, with a numerical reduction in recurrent SCAD. These findings support the safety of a conservative approach and the integration of structured, multidisciplinary SCAD care into clinical practice.

## Supplementary Information

ESM1: Supplementary material 1

## Data Availability

The data underlying this article will be shared on reasonable request to the corresponding author
