# Supplementary material for: Impact of a multidisciplinary clinical pathway on the management of spontaneous coronary artery dissection
Source: Neth Heart J. 2026 May 18;34(6):225–35. doi: 10.1007/s12471-026-02050-w (PMC13216445; doi:10.1007/s12471-026-02050-w)
Supplement: Supplementary file 1 — ESM1: Supplementary material 1 [file 12471_2026_2050_MOESM1_ESM.docx]

|  | **Total (*n* =117)** | **Treated in SCAD care pathway (*n* =63)** | **Not treated in SCAD care pathway (*n* =54)** | **P-value** |
| --- | --- | --- | --- | --- |
| Angiographic screening FMD (%) | 52 (44.4) | 43 (68.3) | 9 (16.7) | **0.006** |
| Angiographic diagnosis FMD (%) | 17 (14.5) | 14 (22.2) | 3 (5.6) | 0.261 |
| CT screening FMD (%) | 42 (35.9) | 29 (46.0) | 13 (24.1) | 0.344 |
| CT diagnosis FMD (%) | 15 (12.8) | 11 (17.5) | 4 (7.4) | 0.838 |
| Location of FMD if present (%) |  |  |  |  |
| - Renal | 13 (11.1) | 11 (20.6) | 2 (3.7) | 0.683 |
| - Carotid | 14 (11.9) | 10 (15.8) | 4 (7.4) | 0.311 |
| - Femoral | 9 (7.6) | 6 (9.5) | 3 (5.6) | 0.137 |

Table 4: Fibromuscular dysplasia distribution

Variables are presented as n (%).

**CT:** computed tomography; **FMD:** fibromuscular dysplasia; **SCAD:** spontaneous coronary artery dissection.
